# The West Africa ICEMR Partnerships for Guiding Policy to Improve the Malaria Prevention and Control

**DOI:** 10.4269/ajtmh.21-1330

**Published:** 2022-10-13

**Authors:** Seydou Doumbia, Mahamoudou Toure, Nafomon Sogoba, Michael Alifrangis, Mahamadou Diakite, Ayouba Diarra, Moussa Keita, Drissa Konaté, Sory I. Diawara, Sidibé M’Baye Thiam, Soumba Keita, Moctar Tounkara, Idrissa Cissé, Vincent Sanogo, Mahamadou H. Magassa, Alyssa E. Barry, Peter J. Winch, Hannah C. Marker, Jeffrey G. Shaffer, Sékou F. Traoré, Günter C. Müller, Liwang Cui, John C. Beier, Jules Mihigo

**Affiliations:** ^1^ Malaria Research and Training Center, University of Sciences, Techniques and Technologies of Bamako, Bamako, Mali;; ^2^ University Clinical Research Center, University of Sciences, Techniques and Technologies of Bamako, Bamako, Mali;; ^3^ Centre for Medical Parasitology, Department of Immunology and Microbiology, University of Copenhagen, Copenhagen, Denmark; Department of Infectious Diseases, Copenhagen University Hospital (Rigshospitalet), Copenhagen, Denmark;; ^4^ Department of Public Health, Faculty of Medicine and Odontostomatology, University of Sciences, Techniques and Technologies of Bamako, Bamako, Mali;; ^5^ National Malaria Control Program, Ministry of Health, Bamako, Mali;; ^6^ School of Medicine, Deakin University, Geelong, Australia;; ^7^ Department of International Health, Johns Hopkins Bloomberg School of Public Health, Baltimore, Maryland;; ^8^ School of Public Health and Tropical Medicine, Tulane University, New Orleans, Louisiana;; ^9^ Division of Infectious Diseases and Internal Medicine, Department of Internal Medicine, University of South Florida, Tampa, Florida;; ^10^ Department of Public Health Sciences, Miller School of Medicine, University of Miami, Miami, Florida;; ^11^ U.S. President’s Malaria Initiative, United States Agency for International Development Office, Bamako, Mali

## Abstract

The Mali National Malaria Control Program (NMCP) recently established a phased set of goals for eliminating malaria in Mali by 2030. Over the past decade, the scale-up of NMCP-led malaria control interventions has led to considerable progress, as evidenced by multiple malariometric indicators. The West Africa International Center of Excellence in Malaria Research (WA-ICEMR) is a multidisciplinary research program that works closely with the NMCP and its partners to address critical research needs for malaria control. This coordinated effort includes assessing the effectiveness of control interventions based on key malaria research topics, including immune status, parasite genetic diversity, insecticide and drug resistance, diagnostic accuracy, malaria vector populations and biting behaviors, and vectorial capacity. Several signature accomplishments of the WA-ICEMR include identifying changing malaria age demographic profiles, testing innovative approaches to improve control strategies, and providing regular reporting on drug and insecticide resistance status. The NMCP and WA-ICEMR partnership between the WA-ICEMR and the NMCP offers a comprehensive research platform that informs the design and implementation of malaria prevention and control research programs. These efforts build local expertise and capacity for the next generation of malaria researchers and guide local policy, which is crucial in sustaining efforts toward eliminating malaria in West Africa.

## BACKGROUND

Mali is a large, semiarid country in west Africa with approximately 20.3 million people.^1^^,^^2^ Over 90% of the population resides in the malaria-endemic areas in the central and southern regions of the country. The Mali National Malaria Control Program (NMCP) has set a goal to eliminate malaria by 2030.^3^ To achieve this goal, the NMCP has planned scaled-up coverage of evidence-based antimalarial interventions targeting 80% to 90% of Mali’s risk population.^3^ These interventions include artemisinin-based combination therapies (ACTs), long-lasting insecticide-treated nets (LLINs), intermittent preventive treatment in pregnancy (IPTp), indoor residual spraying (IRS), and seasonal malaria chemoprevention (SMC). These efforts have yielded substantial declines in malaria morbidity and mortality, with notable improvements evidenced through multiple malariometric indicators.^4^^–^^7^ This progress has been hampered by numerous challenges and policy gaps, many of which were unanticipated. Important gaps in current malaria control policies include insufficient knowledge on the contextual effectiveness of evidence-based preventive treatment strategies (such as SMC and vector control measures such as LLINs and IRS) and limited scientific evidence to support decisions on interventions best suited to the local context for effective malaria control.^8^ For instance, the efficacy of LLINs may vary with the biting behavior of malaria vectors, and the effectiveness of SMC may be affected by the length of malaria transmission seasons. Other gaps include inadequate monitoring of the effectiveness of current malaria control interventions. Significant challenges observed here were shifts in peak malaria transmission seasons, changes in malaria age demographic profile (particularly among children), suboptimal implementation of control interventions, resistance of *Anopheles* mosquito vectors to widely used insecticides,^9^^,^^10^ and the potential emergence of antimalarial drug resistance.^8^^,^^11^^,^^12^

The NMCP and its partners have long prioritized operational and implementation research that applies evidence-based approaches to improve the effectiveness of control interventions. These efforts leveraged research programs through the West Africa International Center of Excellence in Malaria Research (WA-ICEMR) and the University of Sciences, Techniques and Technologies of Bamako (USTTB) to enhance the partnership between researchers and decision-makers to deal effectively with the challenges and policy gaps to malaria control. Launched in 2010, the WA-ICEMR encompasses three research focuses: epidemiology, immunogenomics, and vector ecology.^13^^–^^15^ These focuses are applied using operational, implementation, and translational research approaches and substantial research capacity-building components. The field sites for the WA-ICEMR studies include three ecological settings with variable malaria endemicity levels, seasonality, lengths of transmission, and transmission intensity.^16^^,^^17^ Because of the direct and long-standing relationship between the WA-ICEMR and the NMCP, this work aims to chronicle recent advancements in malaria prevention and control policy in Mali achieved through these collaborations and provide the research community with perspectives for utilizing these approaches in malaria-endemic settings.

### Stakeholder partnerships for addressing the research needs for malaria prevention and control policy.

The WA-ICEMR was designed in response to research questions pertaining to Mali’s high malaria burden despite the extensive deployment of established interventions. The WA-ICEMR works closely with NMCP and its partners, including the U.S. President’s Malaria Initiative (PMI), the Global Fund, and the United Nations Children’s Fund (UNICEF). These coordinated efforts occur through complementary and supportive roles. During the establishment of the WA-ICEMR, study investigators performed a comprehensive needs assessment through in-depth interviews, focus groups, and training workshops. Interviews were conducted with key stakeholders, including country, regional, and district health officers, using iterative approaches to define research priorities. These priorities and other collaborative initiatives are detailed in Table 1.

**Table 1:** Selected NMCP operational and implementation research priorities addressed in collaboration with the WA-ICEMR

Malaria Control Program component*	Operational challenges encountered in implementing malaria control program component	Operational research performed to mitigate operational challenges
Malaria case management and treatment	Lack of adequate and current management and treatment practices at local health facilities	Performance improvement techniques offered to health workers on a regular basis to improve adherence to current clinical treatment guidelines as well as training in malaria diagnosis
Malaria in pregnancy	Low health worker compliance with guidelines for administration of IPTp in health facilities Low coverage and uptake of IPTp with three doses of SP	New service delivery strategies and behavior change interventions to increase SP compliance
SMC	Limited knowledge on level of compliance, side effects and possible barriers to SMC treatment	Post-SMC surveys on compliance and possible SMC-caused side effects and drug metabolite measurement (ELISA) for compliance
	Lack of knowledge on the long-term impact of SMC on malaria epidemiology and transmission (rebound effect on immune response, burden of the disease, parasite genotypes and possible drug resistance development)	SMC impact studies on prevalence and incidence of *Plasmodium falciparum* infection and disease Cohort-based immunogenomics studies to measure the possible impact of SMC on immunological indices Molecular/genomic surveillance of antimalarial drug resistance markers studies and potential impact on malaria incidence
	Need for evaluation of alternative drugs for SMC	Test of DHA-PQ as an alternative drug for SMC
Antimalarial drug resistance	Lack of knowledge on current prevalence and the possible impact of molecular antimalarial drug resistance markers relevant for treatment (ACTs) and preventive treatment	Repeated molecular surveillance of antimalarial drug resistance markers and ex vivo testing
Malaria mosquito transmission	Need for updated knowledge on the impact of current vector control strategies on vector abundance, species composition, and biting behavior	Longitudinal monitoring of entomological parameters at WA-ICEMR study sites
	Lack of knowledge on vector habitat and survival strategies through the dry season	Studies of vector ecology and larval source identification applying drones, ground-truthing and GIS/RS
LLIN use	Lack of updated knowledge on coverage, usage, and residual effects of ITNs on adult mosquitoes	Coverage and usage patterns of ITNs regarding trends in malaria transmission
	Need for evidence on the effectiveness of the deployment of next-generation ITNs	Assessment of the residual effect of insecticides Testing for the effectiveness of next-generation ITNs
Insecticide resistance	Need for regular, routine information on insecticide resistance profiles of primary malaria vectors	Continuous monitoring of susceptibility patterns in vector populations, particularly in intervention areas (molecular resistance markers, genomic surveillance)
Monitoring and evaluation	Large amounts of data at both the individual and molecular levels, with limited capacity for data modeling and analysis	Assistance with analyses for routine data monitoring Application of monitoring data to improve program implementation Data sharing and continuous analyses of research data to assist NMCP

ACT = artemisinin-based combination therapy; DHA-PQ = dihydroartemisinin-piperaquine; ELISA = enzyme-linked immunosorbent assay; GIS = geographic information systems; ITN = insecticide-treated net; IPTp = intermittent preventive treatment of malaria during pregnancy; LLIN = long-lasting insecticide-treated bed net; NMCP = Mali National Malaria Control Program; RS = remote sensing; SMC = seasonal malaria chemoprevention; SP = sulfadoxine-pyrimethamine; WA-ICEMR = West Africa International Center of Excellence in Malaria Research.

* Performed by the Mali NMPC.

The research portfolio for the WA-ICEMR was defined according to feedback from its scientific advisory group and the NMCP and its PMI partners during planning meetings for developing the annual malaria operational plan.^18^ This collaboration facilitated the regular dissemination of research findings through organized scientific conferences and workshops.

### WA-ICEMR research studies and activities supporting NMCP initiatives.

NMCP approaches for reducing malaria morbidity and mortality apply targeted malaria prevention and control interventions according to the epidemiological and entomological profiles of particular geographic zones. Thus, the NMCP relies on frequent, regular, and accurate epidemiological, entomological, and climate data to ascertain the quality and impact of malaria control interventions. The WA-ICEMR is established in different ecological settings and provides a unique opportunity for accurate and real-time data assessment of the temporal and spatial changes in malaria transmission, infection, and disease. It was of interest to quantify these changes before and after launching large-scale interventions such as LLIN distribution and SMC to inform the NMCP regarding its impact and cost-effectiveness. One such initiative that the WA-ICEMR extensively studied was SMC. SMC has been supported by PMI in Mali since 2014^18^ and is guided through operational and implementation research by the NMCP and WA-ICEMR. A host of approaches for improving SMC effectiveness have been evaluated and performed through WA-ICEMR studies.^6^^,^^19^ For example, the WA-ICEMR and the NMCP jointly studied whether the extension of SMC was suitable for children at least 5 years of age (who are not covered by Mali’s current SMC policy) and whether increasing the number of rounds in areas with longer seasonal malaria conditions would yield improved results.^6^^,^^19^^,^^20^ Another SMC implementation study involved a large community trial to assess the effectiveness of SMC for children between 5 and 9 years old using dihydroartemisinin-piperaquine (DHA-PQ) as an alternative treatment strategy to the standard SMC regimen (sulfadoxine-pyrimethamine with amodiaquine [SP-AQ], clinical trial number NCT04149106). Substantial declines in malaria incidence rates were reported for populations covered by DHA-PQ or SP-AQ, and the effectiveness of DHA-PQ in preventing malaria and improving compliance rates among children between 5 and 9 years old became apparent. After these promising results, PMI and NMCP continued to support the implementation of these extended SMC strategies. The WA-ICEMR also addressed several other research questions related to SMC and ACT treatments to guide malaria control policy. These questions focused on malaria pathogenesis, molecular diagnostic testing performance, treatment compliance, parasite genetic diversity, drug resistance markers, tests for detecting counterfeit and substandard artemisinin drugs, and the impacts of interventions on immune responses and potential rebound effects for discontinued SMC use. Through genotyping studies of *Plasmodium falciparum* parasites and whole-genome sequencing elucidating drug resistance markers, the WA-ICEMR quantified temporal changes in parasite diversity in relation to drug pressure in SMC implementation.

Vector control research is yet another topic of collaboration between the WA-ICEMR and NMCP. Vector research focuses on highly complex and heterogeneous patterns of insecticide resistance observed in Mali.^10^^,^^21^^,^^22^ These efforts underscore the importance of continuously monitoring susceptibility profiles and vector population bionomics. The WA-ICEMR addressed several research questions affecting malaria transmission, including the impacts of dry season vector ecology and insecticide resistance.^17^^,^^22^ The WA-ICEMR study sites supported the NMCP by collecting, capturing, and disseminating data on insecticide resistance and trends of malaria vector transmission in response to control interventions. The WA-ICEMR also collaborated with the WHO, resulting in a pilot multisectoral intervention to control malaria vectors that contributed to insecticide resistance mitigation.

Vector ecology studies assist in understanding malaria transmission dynamics, including identifying sources and survival strategies of vector populations during extended dry seasons. Findings from vector ecology studies performed during the dry season provide implementation research opportunities for testing the added value of malaria control strategies targeting dry season transmission, including larval control^23^ and reactive treatment strategies.^24^ Ideally, these interventions should be jointly performed with existing control tools such as LLINs and SMC. Research findings on Insecticide resistance are particularly important in supporting the deployment of the next generation of LLINs.^10^ However, further research in this area is needed to optimize the use of these approaches, as they may have only a modest impact in the presence of mechanisms of pyrethroid resistance (other than metabolic detoxification by oxidase enzymes). Thus, where and when the next-generation LLINs might have a positive impact on malaria transmission remains an important issue, given its relatively high cost and the need for two or more compounds of different insecticide classes to make a single product to combating resistance.^25^ The high levels of outdoor transmission observed in the studies covered here call for additional vector control tools to complement current indoor strategies. Translational research on attractive targeted sugar baits implemented at Mali field sites^26^^,^^27^ through the collaboration between the WA-ICEMR and Innovative Vector Control Consortium (IVCC) also offers new perspectives for improving vector control strategies. The WA-ICEMR has recently planned studies with the NMCP and PMI in more extensive randomized trials to determine the effectiveness of these new approaches.

### Research capacity building supporting malaria control and prevention.

The WA-ICEMR was established to strengthen local research leadership to build sustainable research capacity to guide malaria control strategies. Local Mali researchers lead the WA-ICEMR multidisciplinary research projects in collaboration with international malaria experts. The research studies generated through the WA-ICEMR has also contributed to research career development for numerous junior investigators. For instance, sponsored research provided internship and thesis opportunities for graduate students (at both the master’s and doctoral levels) to address malaria prevention and control research questions. Numerous manuscripts led by Malian junior investigators from these efforts have since been published (Table 2).

**Table 2:** Selected manuscripts led by Malian junior investigators since 2020

Lead author	Year published	Topic
Konate^6^	2021	Seasonal malaria chemoprevention efficacy and community acceptance
Touré^28^	2022	Impact of interventions on malaria trends
Konaté^19^	2020	Seasonal malaria chemoprevention efficacy
Ateba^29^	2020	Space–time trends in malaria incidence
Konaté^30^	2021	Seasonal malaria chemoprevention and nutrition
Ateba^31^	2020	Malaria incidence prediction
Keita^22^	2020	Indoor residual spraying and malaria incidence
Keita^32^	2021	Malaria insecticide resistance
Traore^26^	2020	Attractive toxic sugar baits
Keita^10^	2021	Indoor and outdoor malaria transmission
Diarra^33^	2021	Attractive toxic sugar baits
Traoré^34^	2021	Antimalarial drug prescriptions
Maiga^35^	2021	Artemisinin-based combination therapy efficacy
Diawara^36^	2021	Genetic variants in *Plasmodium falciparum* malaria

The WA-ICEMR also leveraged a host of NIH research training grants, focusing on operational research and bioinformatics and data science approaches to training the next generation of malaria researchers in West Africa. One example is the West Africa Center of Excellence in Bioinformatics Training, which provides enhanced short- and long-term bioinformatics and data science research training and funding for independent pilot projects for students and postdoctoral junior researchers.^37^^,^^38^ Operational and implementation research training workshops are regularly organized with NMCP and partners to identify research questions of interest to control implementation improvement. The workshops organized through the WA-ICEMR research program increase awareness of malaria control program managers and district healthcare officers on the latest research findings and also generate a bank of research questions for graduate students for thesis research and career development to support local health information systems.

### Leveraging WA-ICEMR research capacity to support malaria surveillance and country preparedness for elimination.

Research laboratory capacity for molecular diagnostics and genomics analysis play pivotal roles in supporting the surveillance and country preparedness for malaria elimination.^39^ For instance, molecular and genomics laboratory resources developed in the past decade have contributed to Mali’s response to the COVID-19 pandemic^40^ with support from the WA-ICEMR. As control efforts progress to elimination phases, strong laboratory capacity will be needed to address emerging challenges, particularly in the areas of diagnostics and drug and insecticide resistance identification. Molecular and genomic surveillance can be used to monitor trends in disease burden, detect emerging antimalarial drugs and insecticide resistance, and detect important changes in parasite/vector populations such as deletion of histidine-rich protein 2/3 genes by *P. falciparum* (hrp2/3).

Collaborations with other regional ICEMRs have enhanced the capacity for performing molecular analyses. The WA-ICEMR has leveraged next-generation sequencing platforms through other regional ICEMRs to support the NMCP with genomic surveillance of SMC and ACT drug and insecticide resistance and changes in parasite populations. ELISA techniques used in these studies are now established and fully functional in Mali, allowing for the measurement of active metabolites of amodiaquine drug levels in the blood, which will assist the NMCP in assessing compliance to SMC treatment and detecting counterfeit or substandard artemisinin drugs.^41^^,^^42^ The WA-ICEMR has built laboratory capacity to improve malaria case management, diagnostics, and other routine laboratory analyses (such as hemoglobin levels). Human resources have been expanded, including biologists, where microbiologists and laboratory technicians currently work at each WA-ICEMR study site. Additional clinicians and medical students were also provided through the WA-ICEMR to facilitate field research activities. The presence of a highly skilled research team contributes to improving access to appropriate healthcare and malaria case management, enhancing the outcome of malaria control interventions.

### Information and data sharing supporting malaria surveillance systems.

The WA-ICEMR assists the NMCP’s efforts to monitor and evaluate malaria control interventions, producing comprehensive, real-time research data that complement routine surveillance data of the NMCP’s 15 sentinel sites established across Mali. Data routinely collected on site are shared by the Mali NMCP through the national health information system and at scientific meetings between the research team and local and national health authorities. The WA-ICEMR supported NMCP activities by routinely assisting in SMC and LLIN distribution campaigns, providing real-time data on post-distribution surveys on coverage, compliance, prevalence, and malaria incidence. The robust data management capacity established through the WA-ICEMR used electronic data capture systems, including REDCap (Vanderbilt University, Vanderbilt, TN) and REDCap mobile applications at all WA-ICEMR study sites.^43^ These improvements provided timely and accurate surveillance data supporting Mali’s health information surveillance systems. Data archived using these platforms serve as research data archives for Malian students and will ultimately be made available through the Clinical and Epidemiology Database (ClinEpiDB) resource.^44^

## CONCLUSIONS AND RECOMMENDATIONS

Over the past decade, the scale-up of control interventions has significantly contributed to alleviating the burden of malaria, as evidenced by notable improvements in multiple malariometric indicators with substantial reductions in malaria prevalence and incidence rates. However, the NMCP and its partners must maintain high coverage levels for preventative interventions to sustain control and prevent the resurgence of malaria. The WA-ICEMR/NMCP collaboration highlights the importance of establishing a platform for operational and implementation research, which is critically needed to determine their short- and long-term impact on malaria transmission and inform program activities. The WA-ICEMR research program is comprehensive, generating valuable data-driven evidence necessary for malaria control, and diagnosis. There is a need to expand WA-ICEMR research on human behavioral interventions, which are instrumental in further understanding the results here and are key to achieving malaria control and, ultimately, elimination. The partnerships described here provide highly influential examples for maximizing the engagement among NMCP and its local, national, and international organizations with research communities. Thus, it is critical to empower local researchers while building the next generation of malaria researchers through partnerships to facilitate the uptake of research findings into policy and program activities. The main recommendation following these studies is to build local expertise in malaria-endemic areas such as Mali. Large-scale, multinational efforts and international partners are needed to support continued malaria laboratory and field research, especially studies focused on malaria parasites, mosquito populations, and vector biting behaviors. These approaches are likely the most plausible pathways for achieving long-term malaria elimination goals.
